# Comparable Stride Time Fractal Dynamics and Gait Adaptability in Active Young and Older Adults Under Normal and Asymmetric Walking

**DOI:** 10.3389/fphys.2019.01318

**Published:** 2019-10-25

**Authors:** Scott W. Ducharme, Jane A. Kent, Richard E. A. van Emmerik

**Affiliations:** ^1^Department of Kinesiology, California State University, Long Beach, Long Beach, CA, United States; ^2^Department of Kinesiology, University of Massachusetts Amherst, Amherst, MA, United States

**Keywords:** split-belt treadmill, correlation structure, gait adaptation, physical activity, aging, statistical persistence, variability, detrended fluctuation analysis

## Abstract

Previous research indicates the correlation structure of gait parameters (i.e., fractal dynamics) decreases with age. This decrease is suggested to reflect a reduced capacity for locomotor adaptation in older adults. The purpose of this study was to investigate potential differences between physical activity-matched young and older adults’ fractal dynamics and gait adaptability during unperturbed and asymmetric walking, and to determine if fractal dynamics predict adaptive capacity. Fifteen young (28.9 ± 5.6 years, nine women) and 15 older (64.7 ± 2.7, nine women) adults with similar habitual physical activity levels walked at preferred speed, half of preferred speed, and asymmetrically whereby their dominant and non-dominant legs moved at preferred and half-preferred speed, respectively. Fractal correlations (scaling exponent α) of stride times were assessed through detrended fluctuation analysis, and gait adaptation to asymmetric walking on the basis of lower limb relative phase. Both cohorts displayed similar fractal dynamics at preferred speed and asymmetric walking, while older adults exhibited greater α during slow walking. Both groups exhibited comparable gait adaptation to split-belt walking based on analysis of lower limb relative phase. Fractal dynamics during preferred speed and asymmetric walking was moderately associated with gait adaptation in the young and older adult cohorts, respectively. In these activity-matched groups, there were no age-based reductions in fractal dynamics or gait adaptation, and fractal scaling α was moderately associated with gait adaptation. These findings suggest that stride time fractal dynamics and gait adaptation may be preserved in older adults who habitually perform moderate intensity physical activity.

## Introduction

In recent decades, the study of gait dynamics has evolved from quantifying the magnitude of gait parameter variability to evaluating the structure of this variability. In human locomotion, fluctuations in timing from heel strike to subsequent heel strike of the same foot (i.e., stride time) at short temporal scales are statistically correlated to larger fluctuations at longer scales (Hausdorff et al., 1995, 1996; West and Griffin, 1998). That is, the magnitude of fluctuations increases at longer time scales, and this increase is not random but rather systematic and structured. The signal at any given point exhibits dependence upon previous and future states, and this is known as statistical persistence. When a signal displays statistical persistence, long (or short) stride times tend to be followed by subsequent corresponding long (or short) stride times. This organization is termed “fractal dynamics” and is quantified by the scaling exponent “α.” A larger α is thought to represent an adaptive locomotor system because these correlations across scales may characterize interactions within physiological processes that coordinate to attenuate internal or external perturbations (Delignieres et al., 2006; Delignieres and Marmelat, 2012; Rhea and Kiefer, 2014).

In young healthy adults, fractal dynamics increase when walking faster or slower than preferred walking speed (Hausdorff et al., 1996; West and Scafetta, 2003; Jordan et al., 2007a), but it is unknown how these dynamics also increase in older adults at speeds other than preferred. Moreover, older adults exhibit less structured, more random stride interval fluctuations (Hausdorff et al., 1997; Kobsar et al., 2014). This suggests a reduction in the interactions across spatiotemporal scales, and a corresponding reduction in gait adaptability. Furthermore, older adults who report a history of falls demonstrate further reductions in α compared with healthy older adults (Herman et al., 2005), providing further evidence that fractal dynamics may be a marker of systemic gait capacity. However, it should be noted that other studies have reported no differences between young and older adults (Bollens et al., 2012) or between fallers and non-fallers (Paterson et al., 2011).

Although fractal analyses are thought to quantify locomotor adaptive capacity (Delignieres et al., 2006; Delignieres and Marmelat, 2012; Rhea and Kiefer, 2014), previous studies have not incorporated paradigms that directly probe the system’s adaptability. Split-belt treadmill walking, where one belt moves at a different speed than the other, reveals that participants consistently attempt to maintain or regain symmetry between legs, quantified as deviation from intended leg phasing (Phase_DEV_, i.e., individuals attempt to walk with legs in anti-phase, even when belts are moving at different speeds) or step length symmetry (Dietz et al., 1994; Choi and Bastian, 2007; Bruijn et al., 2012). Older adults adapt their gait patterns less and at a slower rate compared to young adults (Bruijn et al., 2012), as reflected by greater step length asymmetry during split-belt walking, suggesting a lower capacity to adapt. We recently found that stride time α during asymmetric split-belt walking, but not during unperturbed walking, is associated with split-belt walking adaptation in young individuals (Ducharme et al., 2018). However, the extent to which α associates with gait adaptation in older adults is unknown.

The purpose of this study was to compare stride time fractal dynamics (i.e., statistical persistence or α) and gait adaptability (Phase_DEV_) in young and older adults. Because previous studies have noted that habitual physical activity affects traditional gait parameters (Wang et al., 2015; Ciprandi et al., 2017), groups were matched for physical activity via self-report (and later verified by accelerometry). It was hypothesized that, compared with young adults, older adults would exhibit: (1) reduced α at preferred walking speed (PWS); (2) reduced α at half-PWS walking, (3) a greater decrease in α (i.e., become more random) during asymmetric vs. symmetric (PWS) walking, given the evidence that perturbations weaken long-range correlated behavior (Diniz et al., 2011), and (4) reduced gait adaptation (i.e., greater Phase_DEV_) during asymmetric walking. We further hypothesized that, in both the young and older groups, (5) stride time α would be associated with gait adaptation, whereby higher α (i.e., greater statistical persistence) would correlate with better gait adaptation (i.e., lower Phase_DEV_).

## Materials and Methods

### Participants

Fifteen young (21–37 years) and 15 older (61–70 years) healthy men and women volunteered for this study (Table 1). *A priori* sample size estimation determined at least *n* = 11 to be sufficient in determining differences between young and older adults in split-belt adaptation (Choi et al., 2009) and α (Hausdorff et al., 1997). Each participant’s limb dominance was determined by asking which leg they would likely use to kick a ball. As part of the inclusion criteria, all participants self-reported partaking in at least 150 min per week of moderate-to-vigorous physical activity (MVPA), based on a Godin Leisure-Time Exercise questionnaire (Godin and Shephard, 1997). Once enrolled, activity level was confirmed by accelerometry (described below). Finally, all participants completed a Physical Activity Readiness Questionnaire (PAR-Q) and informed consent document. The local Institutional Review Board approved this study.

**TABLE 1:** Subject demographics.

	**Young (*n* = 15)**	**Older (*n* = 15)**	***p*-value**
Sex	9 F, 6 M	9 F, 6 M	–
Age (years)	28.9 ± 5.64	64.7 ± 2.7	–
Height (cm)	169.9 ± 10.3	168.7 ± 9.1	0.73
Body mass (kg)	74.3 ± 10.3	74.9 ± 9.4	0.85
Treadmill preferred walk speed (m⋅s^–1^)	1.17 ± 0.16	1.08 ± 0.16	0.13
Self-report MVPA (min⋅day^–1^)	43.3 ± 17.6	48.9 ± 26.9	0.50
Objective MVPA (min⋅day^–1^)	53.7 ± 17.8	42.8 ± 26.3	0.20

*Data are mean ± SD; MVPA = daily minutes of moderate-to-vigorous physical activity by questionnaire (self-report) and accelerometry (objective).*

### Experimental Apparatus

Participants walked on a split-belt treadmill (Bertec Corporation, Columbus, OH, United States) wearing a shoulder-strapped harness at all times to prevent contact with the ground in the event of a fall. Kinematics were collected using four high speed cameras (Oqus, Qualisys, Gothenburg, Sweden) at 120 Hz. Retro-reflective markers were used to represent bony landmarks and provided 3D spatiotemporal information. A total of eight markers were placed bilaterally on the toe (fifth metatarsal), heel (3 cm inferior to the lateral malleolus), knee (femoral lateral epicondyle), and hip (greater trochanter). These markers were used to create a sagittal plane leg (hip to heel) segment, and to determine gait events.

### Experimental Protocol

This study took place over the course of two sessions. Session 1 first entailed determination of PWS on the treadmill using a modified protocol to that of Jordan et al. (2007b). The tied (i.e., same speed) treadmill belts began at 0.5 ms^–1^ and increased by 0.1 ms^–1^ every 5 s until participants declared the speed to be their preferred or comfortable speed. The treadmill was then increased to a speed 0.3 ms^–1^ greater than their preferred speed and subsequently reduced in speed by 0.1 ms^–1^ every 5 s until participants again declared the speed to be their preferred. The mean of the two values was considered the PWS.

Following a standing calibration, participants walked for 15 min at PWS, and then for 20 min at half-PWS; both trials were followed by a 5-min seated rest on a chair placed upon the treadmill. Finally, participants were exposed to asymmetric, split-belt walking, whereby the non-dominant leg traveled at PWS and the dominant leg shifted between PWS and 75% of PWS five times over the course of 6 min. This asymmetric trial served as a habituation to asymmetric walking, and therefore data were not included in subsequent analyses. During all walking trials, participants were instructed to walk normally, generally near the center of the treadmill, and avoid touching the handrails as much as possible. Following the walking trials, participants were given a hip-worn accelerometer (ActiGraph GT3X, ActiGraph, Pensacola, FL, United States) and a physical activity log. They were instructed to wear the activity monitor for 1 week during all waking hours, except for events involving water such as swimming or bathing.

Session 2 occurred 1 week later. After a standing calibration and 10-min warm-up at the PWS determined during the first session, participants performed a single asymmetric walking trial (split-belt) lasting 12 min. During this trial, the treadmill belt under the dominant leg traveled at PWS, while the belt under the non-dominant leg traveled at half-PWS (i.e., 2:1 ratio). Participants were encouraged to only touch the handrails initially while the treadmill speed ramped up, if needed, and to try to not touch them otherwise.

### Data Analysis

Kinematics were collected using Qualisys Track Manager (Qualisys, Gothenburg, Sweden), and exported to MATLAB (The MathWorks, Natick, MA, United States) for all analyses. Markers were low-pass filtered at 7 Hz using a fourth order zero-lag, dual-pass Butterworth filter. Heel strikes were identified by the maximum peaks in the anterior–posterior (AP) direction of the heel marker (Kang and Dingwell, 2008). Stride time was defined as the timing from heel strike to subsequent heel strike of the same foot. To reduce the effect of one or few errant or atypical stride times (Hausdorff, 2009), we removed any stride times that were >2 times the interquartile range +75th percentile, or <2 times the interquartile range −25th percentile. Of the 90 total trials (i.e., 30 participants each performed three walking trials), most (*n* = 88) were at least 512 strides in length, and thus analysis was truncated to the first 512 strides (Delignieres et al., 2006). Two of the trials contained less than 512 strides (*n* = 496 and *n* = 501 strides). Because these two trials were close to 512, and because the value of 512 is a general guideline and not a strict threshold, these entire data sets were included in the analyses. Minutes per day of MVPA measured by the ActiGraph were ascertained within the ActiLife software (version 6.13.3, ActiGraph, Pensacola, FL, United States) using the Freedson cutpoints (Freedson et al., 1998). The minimum criterion for inclusion of PA data was 3 days with 10 or more hours per day of wear time.

### Determination of Correlation Structure

Stride time fractal dynamics were evaluated using detrended fluctuation analysis (DFA). DFA estimates the correlation structure of time series stride times by quantifying the magnitude of localized fluctuations across various temporal scales. DFA is well described in the literature (Hausdorff et al., 1995, 1996, 2000; Peng et al., 1995). Briefly, a biological signal is integrated and sectioned into non-overlapping windows of length *n*. Within each window, a least-squares linear trend line is fit to the signal, and a root-mean-square analysis quantifies fluctuation magnitude:

(1) $$F\,\left(n\right)=\sqrt{\frac{1}{\,N/n}\,\sum_{j=1}^{\,N/n}\frac{1}{n}\,\sum_{i=1}^{n}{\left(X_{i}-{\bar{X}}_{i}\right)}^{2}}$$

where *F*(*n*) is the fluctuation magnitude at window *n*, *N* is the total number of strides, *X*_*i*_ is the integrated signal at stride interval *i*, and ${\bar{X}}_{i}$ is the y-coordinate location of the local trend within window *n*. *F(n)* is obtained for all non-overlapping windows (*j*) of size *n* (total number of windows = *N/n*), and averaged, yielding a single fluctuation magnitude for each scale size. Window sizes ranged from 4 to N/10, and totaled 15 different window sizes that were evenly spaced in the log domain (Almurad and Delignières, 2016; Liddy and Haddad, 2018). When F(*n*) is plotted against *n* on a double logarithmic plot, the slope of the line of best fit represents the scaling exponent, or α, based on the relationship: *F*(*n*) ≈ *n*^α^. When 0.5 < α ≤ 1.0, the signal exhibits statistical persistence (i.e., a large stride time tends to be followed by another large stride time, and vice versa). When α = 1.0 it represents the so-called 1/f phenomenon (West and Shlesinger, 1990; Diniz et al., 2011), which signifies a behaviorally complex, adaptable system (Lipsitz and Goldberger, 1992; Lipsitz, 2002). When α∼ 0.5, the signal lacks correlated structure and thus reflects poor adaptability. When α < 0.5, the signal exhibits anti-persistence, whereby a large stride time tends to be followed by a smaller stride time, and vice versa. Finally, when α > 1.0, the signal is overly persistent, displays minimal fluctuations from stride to stride, and considered highly constrained and therefore less adaptable (Rhea and Kiefer, 2014).

### Analysis of Gait Adaptation

To quantify the adaptive capacity of the locomotor system, relative phasing between the legs was assessed. Previous studies have indicated leg relative phasing shifts in response to exposure to asymmetric walking, and this shift reverts to resemble unperturbed walking after adaptation (Choi and Bastian, 2007; Choi et al., 2009). Each “leg” was constructed as a segment from the greater trochanter to the ipsilateral heel. The sagittal plane angle was calibrated to the leg angle during upright quiet standing. Each stride was normalized to 100 data points, and a cross correlation function was applied to each stride for the right and left leg angles. During normal, unperturbed walking, individuals display perfect anti-phasing. Thus, gait adaptation was calculated as the deviation from intended phasing (anti-phase, i.e., maximal negative correlation at zero lag) for each stride (Choi and Bastian, 2007). From these data, two variables were calculated. First, the absolute magnitude of deviation from intended phasing (Phase_DEV_) across the first 50 strides was determined. Second, the time-to-adaptation, representing the number of strides required to “settle” or “stabilize” the gait, was ascertained (for details, see Ducharme et al., 2018).

### Statistical Analyses

Demographic variables were compared across cohorts using independent samples *t*-tests. Fractal dynamics (α) across cohorts and conditions were assessed using within-subject, repeated measures analyses of variance (ANOVA), with gait condition as the within-subject factor, and age group as the between-subject factor. The targeted gait condition comparisons were PWS vs. half-PWS for unperturbed walking comparisons, and PWS vs. split-belt for differences between unperturbed and perturbed walking, assessed separately for each leg. Cohort differences in α for each condition and targeted condition comparisons were also assessed using Cohen’s *D* effect size (ES) calculations, with the convention that 0.2, 0.5, and 0.8 represent small, moderate, and large effects, respectively. Cohort differences in gait adaptation (Phase_DEV_ and time-to-adaptation) were evaluated using unpaired *t*-tests and ES calculations. Finally, the relationships between gait adaptation (Phase_DEV_ and time-to-adaptation) and fractal scaling during PWS, half-PWS, and split-belt were determined using linear and quadratic regressions, separately for each group. We also reported standardized regression coefficients (i.e., independent and dependent variables standardized so that variance = 1). Findings were accepted as significant when *p* ≤ 0.05. All statistics were computed using R-Studio (Version 1.0.136, R Foundation for Statistical Computing, Vienna, Austria).

## Results

The study cohorts had the same sex distribution and were not different in height, body mass, PWS, or MVPA (Table 1). The physical activity data indicate that, on average, these were relatively active individuals (Troiano et al., 2008).

**FIGURE 1:**
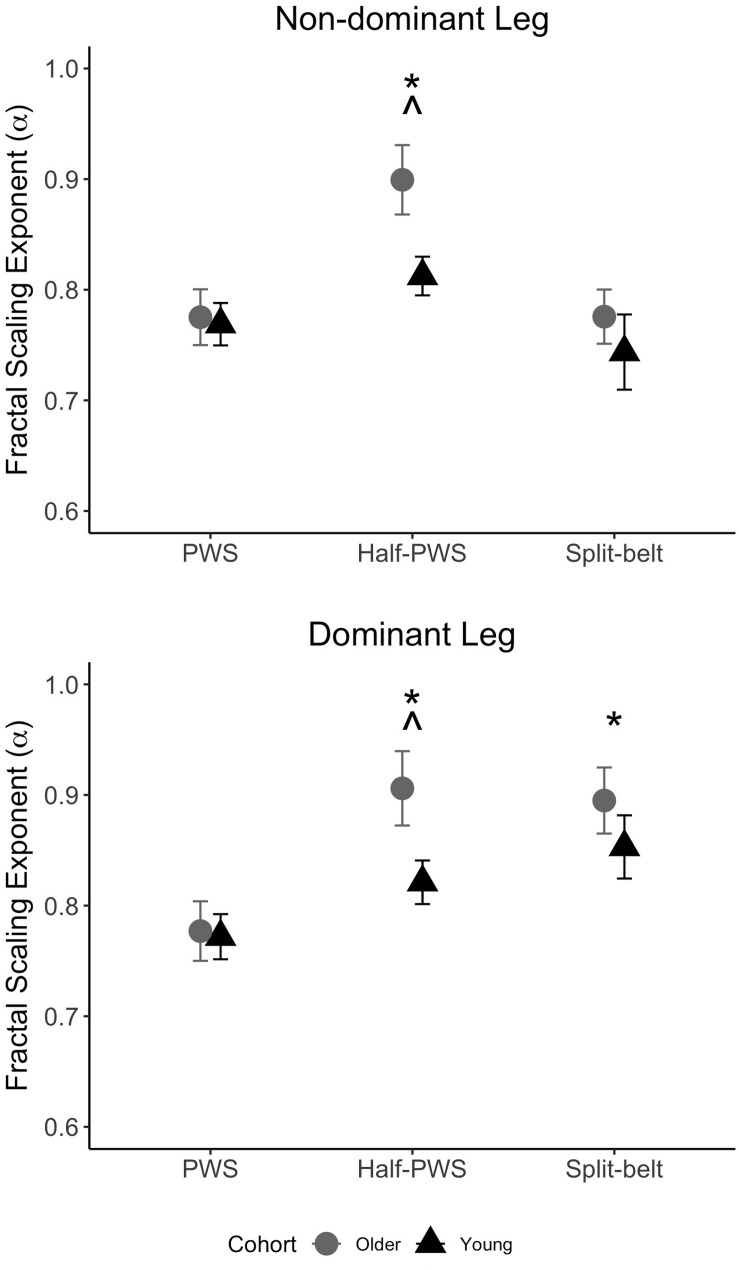
Fractal scaling across conditions for the non-dominant (top) and dominant (bottom) limbs in young (black triangles) and older (gray circles) adults. Data are mean ± SEM. *P* < 0.05: ^∗^ indicates > PWS; ^∧^ indicates condition (PWS, half-PWS) by cohort interaction.

Evaluation of unperturbed walking (PWS vs. half-PWS) revealed young and older adults exhibited similar scaling exponents during PWS, while the older group’s exponents increased more so than that of the young group during half-PWS (Table 2 and Figure 1). There was a main effect of condition (*F*_(__1__,__28__)_ = 20.98, *p* < 0.001 and *F*_(__1__,__28__)_ = 21.35, *p* < 0.001), and an age by condition interaction (*F*_(__1__,__28__)_ = 4.85, *p* = 0.036 and *F*_(__1__,__28__)_ = 4.28, *p* = 0.047) for both the non-dominant and dominant legs, respectively. ES differences between PWS and half-PWS dominant leg α were −0.63 for the young and −1.09 for the older cohort. ES differences between PWS and half-PWS non-dominant leg α were −0.61 for young and −1.13 for the older cohort.

**TABLE 2:** Stride time fractal dynamics (α) and adaptation measures.

		**PWS**	**Half PWS**	**Split-belt**
Stride time-N	Y	0.77 (0.04)	0.81 (0.04)	0.74 (0.03)
	O	0.78 (0.05)	0.90 (0.07)	0.78 (0.05)
	ES	0.07	0.88	0.28
Stride time-D	Y	0.77 (0.04)	0.82 (0.04)	0.85 (0.06)
	O	0.78 (0.06)	0.91 (0.07)	0.89 (0.06)
	ES	0.05	0.80	0.37
Phase deviation	Y			0.20 (0.09)
	O			0.12 (0.07)
	ES			−0.56
Time-to-adaptation (strides)	Y			82.5 (69.2)
	O			44.4 (55.7)
	ES			−0.34

*Data are mean (95% CI); Y = young adults; O = older adults; N = non-dominant leg; D = dominant leg; ES = effect size.*

For asymmetric walking, stride time α during split-belt compared to PWS increased in the faster-moving dominant leg (*F*_(__1__,__28__)_ = 12.30, *p* = 0.002), but not the slower-moving non-dominant leg (*F*_(__1__,__28__)_ = 0.19, *p* = 0.66), for both groups (Figure 1). There was no effect of age nor an age-by condition interaction for either leg (*p*’s > 0.35). For gait adaptation, there was no main effect of age for Phase_DEV_ (*F*_(__5__,__140__)_ = 2.34, *p* = 0.14) or time-to-adaptation (*F*_(__5__,__140__)_ = 0.84, *p* = 0.37, Table 2). Effect size results showed a moderately greater amount of Phase_DEV_ for the younger group (ES = −0.56).

**FIGURE 2:**
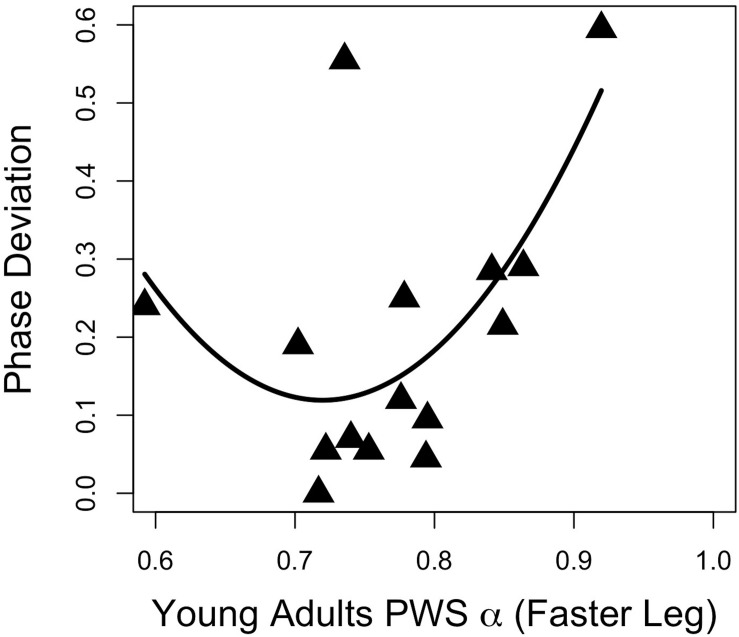
Gait adaptation (Phase Deviation) versus the faster-moving leg’s α during PWS in young adults.

**FIGURE 3:**
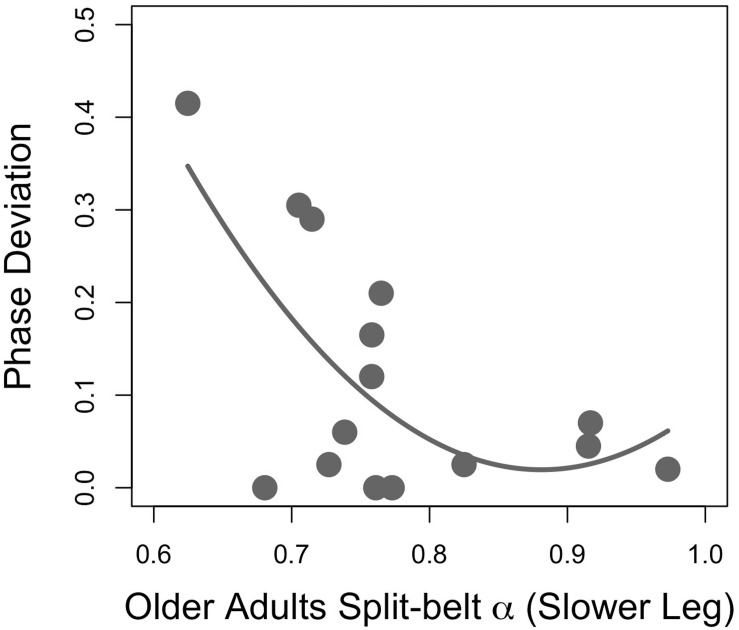
Gait adaptation (Phase Deviation) versus the slower-moving leg’s fractal scaling during asymmetric (split-belt) walking in older adults.

Regression analyses of young adults’ stride time fractal dynamics during PWS and gait adaptation (Phase_DEV_) revealed a moderate quadratic association between the faster-moving leg’s PWS α and phase deviation (Figure 2; *F*_(__2__,__12__)_ = 3.94, *p* = 0.049, *R*^2^ = 0.30, Phase_DEV_ = 9.92α^2^ − 14.29α + 5.26, standardized regression coefficients 7.40α^2^ − 10.65α). All other *R*^2^ values were ≤0.20 and *p*’s > 0.05 for PWS and half-PWS associations with Phase_DEV_ and time-to-adaptation (see Supplementary Table 1). Older adults’ scaling exponents during PWS and half-PWS were not associated with gait adaptation (all *p*’s > 0.05, *R*^2^’s ≤ 0.15, see Supplementary Table 2). However, fractal dynamics during split-belt was moderately associated with Phase_DEV_ in the older group, such that gait adaptation improved as α for the slower-moving leg approached 1.0 (Figure 3; *F*_(__2__,__12__)_ = 4.16, *R*^2^ = 0.31, *p* = 0.042, Phase_DEV_ = 4.98α^2^ − 8.78α + 3.89, standardized regression coefficients 5.71α^2^ − 10.07).

## Discussion

The purpose of this study was to examine potential differences in stride time fractal dynamics and gait adaptability in young and older adults during steady state and asymmetric walking, and to ascertain if fractal dynamics are associated with the adaptive capacity of the human locomotor system. A key feature of this study design was the evaluation of young and older groups of comparable habitual physical activity. Notably, none of hypotheses 1–4 were supported, as these physical activity-matched young and older groups exhibited similar fractal dynamics during unperturbed preferred speed walking (rejecting *hypothesis 1*); α was *greater* in older compared to young during slow-speed walking (rejecting *hypothesis 2*); α *increased* from PWS to split-belt walking in the older group for the faster-moving limb and did not change for the slower-moving limb, similar to the younger group (rejecting *hypothesis 3*); and both cohorts exhibited similar adaptive gait performance (rejecting *hypothesis 4*). *Hypothesis 5* was partially supported in that α in the faster-moving leg was moderately associated with gait adaptation (Phase_DEV_) in young adults, while this adaptation in the older adults was moderately related to α in the slower-moving leg. Overall, these results indicate that fractal dynamics and gait adaptability are largely preserved in older adults with PA levels comparable to those of young adults.

### Preferred Speed Walking Fractality Was Not Different Between Young and Older Adults

Stride time fractal dynamics have been reported to decrease in older vs. young adults (Hausdorff et al., 1997). These findings were interpreted as the manifestation of impaired neurological functionality. In a more recent experiment, age did not impact α (Bollens et al., 2012). One explanation for the lack of age effects was a small sample size; six older adults aged 67–79 years (Bollens et al., 2012) were evaluated. Moreover, the study by Bollens et al. (2012) performed power spectral analysis to evaluate α. Considering power spectral analyses and DFA both essentially evaluate a signal’s contents in terms of power at different frequencies (Hausdorff et al., 1996; Ducharme and van Emmerik, 2018a), the two methods should theoretically yield similar results; perhaps differences in the nuances of each algorithm led to results that conflicted with earlier reports. Specifically, Kiyono (2015) noted that DFA corresponds with power spectral analysis only when the DFA algorithm uses higher order polynomials whereas this study utilized first order linear fitting. This study included younger older adults (61–70 years). Regardless, the current study provides evidence that accounting for physical activity negates apparent age-related differences in PWS stride time α. That is, while healthy yet otherwise sedentary older adults may potentially exhibit reductions in α, participating in regular physical activity may attenuate or eliminate these reductions. To address this question more directly, studies that either assess both physically active and sedentary older adults, or intervene to increase physical activity and determine the effects on α, are needed.

### Fractal Scaling Was Greater During Slow Walking

Slow walking has been shown to yield higher α values compared to preferred-speed walking (Hausdorff et al., 1996; Jordan et al., 2007b), and the results from this study further support this notion (Figure 1). Interestingly, the older adults’ α increased more than that of the young adults. Considering that an α closer to 1.0 (i.e., 1/f) represents greater adaptive capacity, these findings suggest that the older adults had a more adaptive gait than the young adults during slow walking. Alternatively, constraints and task difficulty appear to impact α. Walking slowly increases older adults’ gait variability magnitude (step length, width and time, swing, stance, and dual support time) compared to young adults (Almarwani et al., 2016). Indeed, slow walking has been described as an overall more challenging gait (Almarwani et al., 2016). A higher α in older adults during the slow walking condition may further indicate that slow walking is more challenging for this group. That is, slow walking should correspond with a large increase in α only if the task is perceived as challenging and requiring a shift to adaptive gait. Indeed, slow walking may be a better test for underlying gait impairments or pathology (Almarwani et al., 2016).

### Fractal Dynamics Increased Comparably in Both Groups During Asymmetric Gait

Corresponding to previous observations in young adults during split-belt treadmill walking (Ducharme et al., 2018), α increased for both cohorts in the faster-moving dominant limb when exposed to asymmetric gait. Constraining the locomotor system via tasks other than steady state preferred speed walking yields fractal dynamics that increase closer to α = 1.0. This shifting of α may be the manifestation of modified interactivity of processes across the various temporal scales being investigated. If persistent fluctuations aid adaptation, altered α represents an emergent and beneficial reorganization of the locomotor system that provides a better ability to respond to task stressors and shift to more stable states.

The increase in α during split-belt walking was not observed in the slower-moving limb, which contrasts the previous observation (Ducharme et al., 2018) of increased α of the slower moving leg during constrained walking in young adults. The main difference between this and the previous protocol was that in the current study participants wore a body-supporting harness. This may have been perceived as an additional constraint because the harness partially limited total body displacement in the anterior–posterior directions. This additional constraint, while allowing the locomotor system to maintain persistence of the faster-moving leg, may have restricted the variance in stride times (e.g., reduced range) in the slower-moving leg. Moreover, the harness may have provided additional information from cutaneous receptors or proprioceptors that could have altered gait in some manner. However, the addition of a harness has been shown to not have any effect on α (Stout et al., 2016).

The lack of differences in α during asymmetric walking between the young and older groups was unexpected but promising. That is, the participant pool consisted of active young and older adults. The previously observed reductions in α in older adults were considered a result of age-related neurological decrements. This loss in neurological function may in fact be a consequence of disuse or underuse that often characterizes physical activity behavior of older adults (Crespo et al., 1996; Brach et al., 2004). Recruiting healthy, active adults aged 61–70 years minimized the potential effects of differences in habitual physical activity or mobility dysfunction on study variables. With the new information provided by this study, we are now well positioned to extend this work to the investigation of potential age-related differences in performance or α to examine the effects of sedentary behavior, poor mobility, and older age (>70 years).

### Young and Older Adults Exhibited Similar Adaptive Performance

Older adults exhibited similar, or perhaps even moderately better, gait adaptation to that of the young group during split-belt treadmill walking. Previous research suggests gait adaptability is lower in older compared to young adults (Bruijn et al., 2012). However, key participant characteristics such a habitual physical activity were not accounted for. Considering that ∼60% of older adults report little or no regular moderate or vigorous physical activity (Crespo et al., 1996; Brach et al., 2004), the older adults in these studies were likely more sedentary than the young adults, placing physical activity as a confounding factor. Thus, previously observed discrepancies between young and older adults (Bruijn et al., 2012) may have been based, at least in part, on the possibility that the older adults recruited were also more sedentary, and/or had been sedentary for a longer time (e.g., the past 30 vs. 5 years). Alternatively, our older cohort was relatively young (61–70 years), and it may be that age-related changes in adaptive performance are more evident at older ages.

### Relationship Between Fractal Dynamics and Gait Adaptation

Recent work (Ducharme et al., 2018) has provided evidence that steady state α during unperturbed walking does not predict adaptive gait performance. The current study indicated a quadratic relationship between young adults’ PWS α and asymmetric walking gait performance (Figure 2). This relationship suggests that those individuals whose unperturbed walking α was ∼ 0.7–0.75 were the most adaptable, as these individuals ultimately exhibited better adaptation. It should be noted that this relationship was moderate. In addition, these findings were not observed in the slower-moving limb, nor in either limb for the older adults, nor in either limb in the previous study (Ducharme et al., 2018). Clearly, more research is needed in order to clarify the relationship between unperturbed walking α and adaptive gait, and would likely benefit from a larger sample size in performing regression analyses.

One question that often arises is that if α∼ 1.0 represents highly adaptive systems, why young, healthy adults don’t exhibit α∼ 1.0 during walking at preferred speed. One explanation is that individuals with values closer to 0.5 or 1.0 are overly random or overly correlated, respectively, and that healthy adults exhibit values that are between these two extremes (Hausdorff, 2007; Rhea and Kiefer, 2014). As an alternative, we argue that unperturbed, steady state walking entails minimal constraints on the locomotor system, and therefore healthy individuals are not sufficiently challenged to display α∼ 1.0 (Ducharme and van Emmerik, 2018a). While α∼ 1.0 may optimize adaptation, other system functions (e.g., metabolic cost of walking) may not be optimized.

In this study, older adults displayed a relationship between asymmetric walking α and gait adaptation similar to that previously observed in young adults (Ducharme et al., 2018). Those whose slower-moving leg’s fractal dynamics increased close to α∼ 0.9–1.0 exhibited the best gait adaptation (Figure 3). These findings may provide further evidence for the potential functional advantages of α in responding to task or environmental stressors. The reduction in α in some individuals may be a result of more large-scale, error-correcting behavior at short temporal scales that manifests as decreased scaling exponents. However, it should again be noted that this relationship was not observed in the older adult’s faster-moving dominant limb, or in either limb for the young adults. The lack of association may be because physically active participants were recruited. Moreover, the absence of associations could have been a result of the inherent variance of biological systems, or a result of other unknown factors. Even the older adults’ α explained only a relatively small portion of the observed variance, indicating other factors must be responsible for the emergent behavior.

### Limitations and Future Directions

Sample sizes for each cohort were *n* = 15. This sample size estimation was based on data from previous studies that reported age cohort differences in both split-belt adaptation and α. However, this number of observations may have not been large enough to comprehensively assess relationships between α and gait adaptation from the regression analyses. To our knowledge, this was the first study to assess age-related associations between α and adaptation on a split-belt treadmill. As such, we could not utilize data from earlier research with similar protocols and analyses. Future studies should incorporate larger number of participants when attempting to understand the relationship between α and gait adaptation.

This protocol consisted of 47 min of walking, including 12 min of asymmetric walking, and as such fatigue may have become a factor. However, PWS and half-PWS trials were separated by a 5-min seated rest, and the split-belt condition occurred 1-week later after a 10-min warm-up walk at a comfortable speed. Moreover, in our previous study (Ducharme et al., 2018), participants performed 75 min of walking, including 45 min of split-belt walking. Rating of perceived exertion was collected in the last minute of each trial. Although not reported, participants generally regarded their exertion as light to moderate intensity. Finally, in this current study we recruited healthy and highly physically active adults, thereby further minimizing the potential for issues with fatigue.

This study assessed fractal dynamics from a monofractal perspective, i.e., a single scaling exponent α can be used to represent the signal. Previous studies report that gait variability may exhibit slightly multifractal characteristics, i.e., the signal requires a spectrum of scaling exponents to fully capture the evolution of the signal (Struzik, 1999, 2000; West and Scafetta, 2003, 2005; Ihlen and Vereijken, 2014). While we previously reported on the multifractal nature of unperturbed and asymmetric gait in young adults (Ducharme and van Emmerik, 2018b), future studies should also investigate the nature of fractal fluctuations in older adults during asymmetric walking.

## Conclusion

The lack of difference in fractal dynamics between active young and older adults during unperturbed walking at preferred speed suggests that an active lifestyle may mitigate previously reported age-based changes in fractal dynamics. Importantly, the greater increase in α in our physically active older compared to the younger cohort suggests a functional adaptation to a gait speed different than preferred that may represent a relatively greater challenge or constraint for older adults. Both cohorts exhibited a similar increase in α during asymmetric walking in the faster-moving but not the slower-moving leg compared to preferred speed walking, a finding consistent with the notion that greater gait challenges require increased fractal correlations. These results indicate the active older adults reorganized their stride timing fluctuations in a similar and beneficial manner as the young adults to ultimately optimize gait adaptability. Finally, the moderate association in young and older adults between unperturbed and asymmetric α with gait adaptation suggests that the emergence of higher fractal scaling enhances (or, at least, associates with) gait adaptation. Future research should evaluate this potential relationship using a full range of gait adaptation paradigms.

## Data Availability Statement

The datasets generated for this study are available on request to the corresponding author.

## Ethics Statement

The studies involving human participants were reviewed and approved by the Institutional Review Board, University of Massachusetts Amherst. The participants provided their written informed consent to participate in this study.

## Author Contributions

SD and RE designed the study. SD, JK, and RE provided updates to the original protocol and interpretation of the study findings, and approved the final version of the manuscript. SD recruited participants, collected and analyzed the data, and drafted the manuscript. JK and RE provided feedback for subsequent revised drafts.

## Conflict of Interest

The authors declare that the research was conducted in the absence of any commercial or financial relationships that could be construed as a potential conflict of interest.
