# Supplementary material for: Comparable Stride Time Fractal Dynamics and Gait Adaptability in Active Young and Older Adults Under Normal and Asymmetric Walking
Source: Front Physiol. 2019 Oct 25;10:1318. doi: 10.3389/fphys.2019.01318 (PMC6823242; doi:10.3389/fphys.2019.01318)
Supplement: Supplementary file 1 [file Table_1.docx]

##### Supplementary Table 1: Association between young adults’ gait adaptability performance and fractal scaling exponents.

| Dependent Variable | Independent Variable | Model | *p* | R^2^ | Significant |
| --- | --- | --- | --- | --- | --- |
|  |  |  |  |  |  |
| Phase_DEV_ at 1^st^ Split-Belt Condition – Young Adults | α_D_ at PWS | Linear | 0.17 | 0.07 |  |
|  |  | Quadratic | 0.049 | 0.30 | * |
|  | α_N_ at PWS | Linear | 0.24 | 0.03 |  |
|  |  | Quadratic | 0.10 | 0.20 |  |
|  | α_D_ at Half-PWS | Linear  Quadratic | 0.77  0.91 | -0.07  -0.15 |  |
|  | α_N_ at Half-PWS | Linear  Quadratic | 0.85  0.75 | -0.07  -0.11 |  |
|  | α_D_ at Split 1 | Linear | 0.17 | 0.08 |  |
|  |  | Quadratic | 0.22 | 0.09 |  |
|  | α_N_ at Split 1 | Linear | 0.20 | 0.06 |  |
|  |  | Quadratic | 0.28 | 0.06 |  |
| TtA at 1^st^ Split-Belt Condition – Young Adults | α_D_ at PWS | Linear | 0.15 | 0.09 |  |
|  |  | Quadratic | 0.11 | 0.20 |  |
|  | α_N_ at PWS | Linear | 0.19 | 0.06 |  |
|  |  | Quadratic | 0.22 | 0.10 |  |
|  | α_D_ at Half-PWS | Linear  Quadratic | 0.99  0.99 | -0.08  -0.16 |  |
|  | α_N_ at Half-PWS | Linear  Quadratic | 0.92  0.59 | -0.08  -0.07 |  |
|  | α_D_ at Split 1 | Linear | 0.19 | 0.06 |  |
|  |  | Quadratic | 0.16 | 0.14 |  |
|  | α_N_ at Split 1 | Linear | 0.19 | 0.06 |  |
|  |  | Quadratic | 0.15 | 0.15 |  |

Note: α_D_ and α_N_ = dominant and non-dominant leg scaling exponents, respectively. TtA = time to adaptation. PWS = preferred walking speed; Half-PWS = half preferred walking speed; Split 1 = first asymmetric split-belt trial, in which the dominant and non-dominant legs moved at PWS and Half-PWS, respectively.
