# Supplementary material for: Comparable Stride Time Fractal Dynamics and Gait Adaptability in Active Young and Older Adults Under Normal and Asymmetric Walking
Source: Front Physiol. 2019 Oct 25;10:1318. doi: 10.3389/fphys.2019.01318 (PMC6823242; doi:10.3389/fphys.2019.01318)
Supplement: Supplementary file 2 [file Table_2.docx]

##### Supplemental Table 2: Association between older group’s gait adaptability performance and fractal scaling exponents

| Dependent Variable | Independent Variable | Model | *p* | R^2^ | Significant |
| --- | --- | --- | --- | --- | --- |
| Phase_DEV_ at 1^st^ Split-Belt Condition – Older Adults | α_D_ at PWS | Linear | 0.53 | -0.04 |  |
|  |  | Quadratic | 0.80 | -0.12 |  |
|  | α_N_ at PWS | Linear | 0.76 | -0.07 |  |
|  |  | Quadratic | 0.94 | -0.15 |  |
|  | α_D_ at Half-PWS | Linear  Quadratic | 0.89  0.37 | -0.08  0.01 |  |
|  | α_N_ at Half-PWS | Linear  Quadratic | 0.83  0.60 | -0.07  -0.06 |  |
|  | α_D_ at Split 1 | Linear | 0.12 | 0.11 |  |
|  |  | Quadratic | 0.26 | 0.07 |  |
|  | α_N_ at Split 1 | Linear | 0.046 | 0.22 | * |
|  |  | Quadratic | 0.04 | 0.31 | * |
| TtA at 1^st^ Split-Belt Condition – Older Adults | α_D_ at PWS | Liner | 0.99 | -0.08 |  |
|  |  | Quadratic | 0.91 | -0.15 |  |
|  | α_N_ at PWS | Linear | 0.80 | -0.07 |  |
|  |  | Quadratic | 0.94 | -0.15 |  |
|  | α_D_ at Half-PWS | Linear  Quadratic | 0.23  0.29 | 0.04  0.05 |  |
|  | α_N_ at Half-PWS | Linear  Quadratic | 0.22  0.31 | 0.04  0.04 |  |
|  | α_D_ at Split 1 | Linear | 0.06 | 0.19 |  |
|  |  | Quadratic | 0.04 | 0.33 | * |
|  | α_N_ at Split 1 | Linear | 0.10 | 0.14 |  |
|  |  | Quadratic | 0.19 | 0.11 |  |

Note: α_D_ and α_N_ = dominant and non-dominant leg scaling exponents, respectively. TtA = time to adaptation. PWS = preferred walking speed; Half-PWS = half preferred walking speed; Split 1 = first asymmetric split-belt trial, in which the dominant and non-dominant legs moved at PWS and Half-PWS, respectively. TtA initially indicated a significant association with α_F_ at Split 1 using a quadratic model (*p* = 0.04, R^2^ = 0.33). However, this relationship was highly influenced by a single outlier (TtA = 400, i.e., he/she did not adapt). Removing this individual erased the association (*p* = 0.25).
